# Effect of Oseltamivir Use on Follow-Up Stroke Mortality

**DOI:** 10.3390/ph18060796

**Published:** 2025-05-26

**Authors:** Pei-Hua Chuang, Bor-Show Tzang, Chih-Chen Tzang, I-Ying Kuo, Chun-Yu Lin, Tsai-Ching Hsu

**Affiliations:** 1 Institute of Medicine, Chung Shan Medical University, Taichung 402, Taiwan; dylan.ymu@gmail.com (P.-H.C.); bstzang@csmu.edu.tw (B.-S.T.); 2 Department of Biochemistry, School of Medicine, Chung Shan Medical University, Taichung 402, Taiwan; 3 Department of Clinical Laboratory, Chung Shan Medical University Hospital, Taichung 402, Taiwan; 4 Immunology Research Center, Chung Shan Medical University, Taichung 402, Taiwan; 5 School of Medicine, College of Medicine, National Taiwan University, Taipei City 100, Taiwan; jerrytzang@gmail.com; 6 Department of Biotechnology, College of Biomedical Science, Kaohsiung Medical University, Kaohsiung 807, Taiwan; iyingkuo@kmu.edu.tw; 7 School of Medicine, Chung Shan Medical University, Taichung 402, Taiwan; 8 Division of Allergy, Immunology, and Rheumatology, Department of Internal Medicine, Kaohsiung Veterans General Hospital, Kaohsiung 813, Taiwan

**Keywords:** influenza infection, oseltamivir, stroke, mortality, retrospective cohort study

## Abstract

**Background/Objectives**: Evidence has indicated an increased risk of stroke in individuals with influenza infection, and the administration of Oseltamivir revealed a lower stroke risk in these individuals. However, the impacts of Oseltamivir and stroke remain limited. **Methods**: The data used in this retrospective cohort study were extracted from the Taiwan National Health Insurance Research Database (NHIRD), which included 281,420 Oseltamivir users and 13,394,652 patients between 1 January 2009 and 31 December 2018. The Oseltamivir group was younger (age 40.1 ± 15.3 years) and had a lower prevalence of comorbidities compared to the non-user group (age 46.2 ± 16.0 years). Stroke incidence and mortality were compared using multivariable Cox proportional hazards models. **Results**: We compared the incidence of ischemic stroke among individuals without a history of ischemic stroke, stratified by Oseltamivir use. After adjusting for age, sex, and comorbidities, Oseltamivir use was not associated with a significantly different risk of stroke (adjusted HR = 1.02, 95% CI: 0.96–1.08; *p* = 0.511). The mortality among individuals with a history of ischemic stroke, being Oseltamivir users (n = 2502), exhibited a lower cumulative mortality rate compared to non-users (4.08% vs. 6.45%). The association remained significant after multivariable adjustment, with an adjusted hazard ratio for mortality of 0.74 (95% CI: 0.61–0.89; *p* = 0.002). **Conclusions:** In this large population-based cohort of patients without a history of ischemic stroke, Oseltamivir use during influenza infection was not associated with stroke incidence after adjusting for age, sex, and comorbidities. Notably, Oseltamivir use in patients with a history of ischemic stroke was associated with reduced all-cause mortality, suggesting a potential survival benefit that warrants further investigation.

## 1. Introduction

Stroke is a critical health concern globally, arising when the blood supply to the brain is abruptly interrupted or reduced, leading to severe neurological impairment. In 2021 alone, stroke affected millions, with 93.8 million survivors, 11.9 million new cases, and 7.3 million fatalities worldwide [1]. Stroke ranks as the third leading cause of death, following ischemic heart disease and COVID-19, and stands as the fourth-highest cause of disability-adjusted life years (DALYs) [1]. Stroke is generally classified into two types, including ischemic stroke, which makes up the majority (approximately 60–80%), and hemorrhagic stroke [2,3]. Transient ischemic attacks (TIAs) or mini-strokes differ from these primary categories, as they involve only brief interruptions in blood flow, usually lasting less than five minutes, and can act as warning signs for future strokes [4]. The impact and burden of post-stroke are profound, encompassing a broad spectrum of physical, cognitive, emotional, social, and financial challenges. Stroke survivors face not only the immediate consequences of the stroke itself but also long-term difficulties that can affect their quality of life [5].

Oseltamivir phosphate (Oseltamivir), commercially known as Tamiflu^®^, was approved in late 1999 as a targeted anti-influenza treatment. As a neuraminidase inhibitor, Oseltamivir effectively and selectively halts the replication of influenza A and B viruses [6]. The action of Oseltamivir is to inhibit the enzymatic activity of viral neuraminidase, an essential enzyme that the influenza virus requires to release new viral particles from infected cells. By blocking this enzyme, Oseltamivir reduces the spread of the virus within the body, helping to shorten the duration of flu symptoms, lessen their severity, and prevent complications. Early administration of Oseltamivir is essential for optimal effectiveness in treating and preventing influenza infections [7,8]. Indeed, clinical evidence has demonstrated that Oseltamivir can significantly reduce the duration of clinical symptoms, lower the risk of complications in the lower respiratory tract, and decrease hospitalizations among patients with natural influenza infection [9,10,11]. Interestingly, recent studies have shown that Oseltamivir is also effective in treating conditions beyond influenza, particularly in treating cancers [12]. Similar findings were observed in recent research that the combination of aspirin, Oseltamivir, and gemcitabine significantly upended pancreatic cancer cell viability and progression [12]. Additionally, a database study suggested that Oseltamivir may have off-label anticancer effects, with evidence showing lower cancer incidence and reduced mortality among Oseltamivir users, particularly for liver cancer [13], supported by in vitro studies indicating mechanisms such as apoptosis or autophagy [14]. A recent large-scale cohort study also reported that Oseltamivir use was associated with a modest but statistically significant increase in the risk of Type 2 diabetes [15].

Influenza and other acute infections have been associated with an increased risk of cardiovascular complications, such as ischemic stroke [16,17,18]. Seasonal patterns indicate a higher incidence of cardiovascular events during the winter, coinciding with periods of heightened influenza activity [17]. Additionally, prior research has demonstrated that receiving an influenza vaccine can reduce the likelihood of cardiovascular events and stroke [19,20,21]. Recently, some studies have established a link between Oseltamivir and cardiovascular disease, as well as stroke. In a retrospective study that enrolled 37,482 cardiovascular patients diagnosed with influenza, lower stroke events were observed in the individuals with Oseltamivir treatment [22]. Another review article has indicated that acute influenza infections can temporarily increase the risk of ischemic stroke. Oseltamivir administration may reduce the stroke risk in the six months following influenza infection [23] Additionally, an elevated plasma N-acetylneuraminic acid level was linked to a higher risk of cardiovascular death (HR 2.11, *p* < 0.001) in heart failure patients who died of cardiovascular-related deaths, accompanied by upregulated neuraminidase expression in their cardiac tissues [24]. Although these findings indicated a link between Oseltamivir and stroke, the impact of Oseltamivir use on stroke risk remains limited. Therefore, we conducted a large-scale retrospective cohort study to investigate correlations between Oseltamivir and stroke development.

## 2. Results

### 2.1. Participant Characteristics

**Figure 1:**
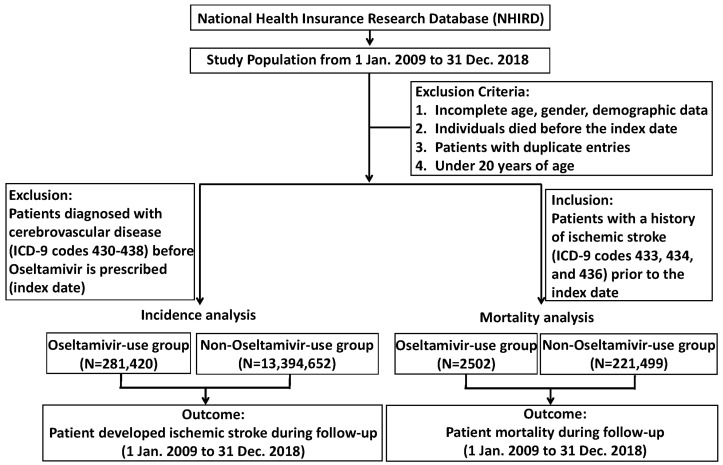
Flowchart of participants.

Figure 1 shows a flowchart of the study participants. Table 1 presents the demographic information of the study population, comprising a total of 13,676,072 individuals. Of these, 281,420 individuals were categorized in the Oseltamivir-use group, while the remaining 13,394,652 were included in the non-Oseltamivir-use group. The analysis showed that 44.8% of the Oseltamivir-use group was male, compared to 46.3% of the non-Oseltamivir-use group. The mean age in the Oseltamivir-use group was 40.1 ± 15.3 years, while in the non-Oseltamivir-use group, it was 46.2 ± 16.0 years. Statistically significant differences in comorbidity prevalence were observed between the two groups. The non-Oseltamivir-use group showed a higher prevalence of hypertension (HTN) (17.1% vs. 10.7%), diabetes mellitus (DM) (8.52% vs. 5.59%), and chronic renal insufficiency (CRI) (1.38% vs. 1.21%), as well as increased rates of peripheral arterial disease (PAD), hyperlipidemia, atrial fibrillation (AF), and coronary artery disease (CAD), all with statistical significance (*p* < 0.001). Conversely, chronic obstructive pulmonary disease (COPD) was more prevalent in the Oseltamivir-use group (3.35% vs. 2.54%).

### 2.2. Comparison of Ischemic Stroke Incidence and Cox Proportional Hazards Analysis

Table 2 compares ischemic stroke incidence and follow-up durations between the Oseltamivir-use group (281,420 patients) and the non-Oseltamivir-use group (13,394,652 patients). The cumulative incidence of ischemic stroke over the follow-up period was lower in Oseltamivir users, at 0.47%, compared to 0.7% in the non-Oseltamivir group. The Oseltamivir group’s crude HR for ischemic stroke was 0.62 (95% CI: 0.59–0.65; *p* < 0.001). After adjusting for sex and age, the HR increased to 1.13 (95% CI: 1.07–1.20; *p* < 0.001). However, with further adjustment for comorbidities, the HR was 1.02 (95% CI: 0.96–1.08; *p* = 0.511), indicating no significant difference in stroke risk after full adjustment.

### 2.3. Comparison of Mortality Rates and Cox Proportional Hazards Analysis

Table 3 provides a comparison of mortality rates and Cox proportional hazards analysis results between the Oseltamivir-use group (2502 patients) and the non-Oseltamivir-use group (221,499 patients), all of whom had a history of ischemic stroke (ICD-9-CM codes 433, 434, and 436) prior to the index date. During the follow-up period (2009–2018), the cumulative mortality rate was significantly lower in the Oseltamivir group, with 102 deaths (4.08%), compared to 14,290 deaths (6.45%) in the non-Oseltamivir group. The Cox proportional hazards analysis also supports the survival benefit associated with Oseltamivir use. The crude HR for mortality was 0.72 (95% CI: 0.59–0.87; *p* = 0.001). This reduction remained statistically significant after adjusting for sex and age (HR 0.75, 95% CI: 0.62–0.92; *p* = 0.005) and further adjustment for comorbidities (HR 0.74, 95% CI: 0.61–0.89; *p* = 0.002). These findings underscore a substantial and consistent mortality benefit linked to Oseltamivir use, even after accounting for potential confounders.

## 3. Discussion

This retrospective cohort study compared stroke incidence, mortality, and comorbidities between Oseltamivir users and non-users. Non-users had a higher prevalence of comorbidities such as hypertension, diabetes, and coronary artery disease, whereas chronic obstructive pulmonary disease was more frequently observed among Oseltamivir users. Although Oseltamivir users initially showed a lower stroke incidence after adjusting for sex and age, this difference became insignificant after adjusting for comorbidities. However, Oseltamivir users demonstrated a significantly lower mortality rate, which remained robust after adjustment for sex, age, and comorbidity. Despite no effect on stroke incidence, this study uniquely highlights Oseltamivir’s association with reduced mortality.

Influenza infection is strongly linked to cardiovascular morbidity [25,26]. It triggers systemic inflammation by elevating proinflammatory cytokines, promoting fibrin deposition, enhancing platelet aggregation, and impairing endothelial function while simultaneously diminishing the anti-inflammatory properties of high-density lipoprotein [27,28,29]. This association underscores the importance of monitoring cardiovascular health in influenza patients [30]. Moreover, stroke has also been linked to influenza infection. A case-crossover analysis based on the Californian population found influenza-like illness to be a potential stroke trigger (OR: 2.88, 95% CI: 1.86–4.47). Supporting evidence from case-control studies further suggests that influenza may precede stroke events, potentially accounting for the increased incidence of stroke observed during winter months. Although the precise mechanisms remain unclear, it is hypothesized that the influenza virus contributes to a prothrombotic state by activating coagulation pathways and impairing natural anticoagulant functions, thereby increasing the risk of stroke or death [31,32,33,34]. Building upon previous findings that suggest that influenza vaccination and Oseltamivir use may reduce stroke risk [23], our study further shows that Oseltamivir use is associated with significantly lower mortality in stroke patients, suggesting its potential role in improving stroke outcomes rather than primary stroke prevention. Overall, these findings may provide a possible explanation for the observation in this study that Oseltamivir users exhibited significantly lower mortality.

Oseltamivir, a key agent in influenza management, is a neuraminidase inhibitor that reduces symptoms and illness duration by inhibiting the cleavage of sialic acid residues [35], thereby lowering the infectivity of the influenza virus [36]. Patients treated with Oseltamivir showed reduced proinflammatory markers, including IL-6, TNF-α, and INF-γ [37,38]. Notably, prophylactic use of Oseltamivir for approximately 10 days in patients on warfarin resulted in a significant increase in the international normalized ratio, suggesting a reduced propensity for clot formation in blood vessels [39]. This finding aligns with observations of reduced mortality in patients treated with Oseltamivir, potentially due to its effects on vasculopathy. Viral infections, including influenza, are frequently associated with vasculopathy, as they trigger inflammatory responses and disrupt endothelial function, ultimately contributing to vascular stenosis, ischemia, and plaque formation [40]. The findings could explain why viral infections might trigger strokes and suggest that taking Oseltamivir may counteract the effects of influenza and reduce stroke-related mortality.

Epidemiological evidence highlights a significantly higher incidence of stroke among individuals with influenza infection [40]. A 2009 retrospective cohort study analyzed data from a large U.S. insurer to evaluate the impact of Oseltamivir use on stroke or TIA within six months following influenza [41,42]. The study found a 28% reduction in the risk of stroke or TIA among Oseltamivir users (HR 0.72; 95% CI 0.62–0.82). However, upon examining a larger and more contemporary population compared to earlier research, the current study did not identify a significant effect of Oseltamivir on stroke incidence after comprehensive adjustments. Several factors could potentially explain this difference. First, stroke incidence is influenced by a range of variables, including geographic location, age, sex, and underlying health conditions, especially hypertension, diabetes mellitus, hyperlipidemia, smoking, and prior stroke or transient ischemic attacks, obesity, and insufficient physical activity [43,44]. The multifactorial nature of stroke makes it difficult to definitively establish the role of Oseltamivir in its prevention. Ethnic differences may also contribute to the variation in findings, as numerous studies have reported disparities in stroke incidence and outcomes between Asian and non-Asian populations [45], whereas our analysis focused on an Asian cohort. For instance, the study by Madjid et al. primarily examined Caucasian individuals [41]. Additionally, differences in study periods may have influenced the findings. Our analysis, conducted from 2009 to 2018, reflects a more recent timeframe compared to the earlier study, which covered the years 2000 to 2006. These temporal and demographic distinctions underscore the need for further research to clarify the potential impact on reducing stroke risk across diverse populations.

While our analysis found a statistically significant association between Oseltamivir use and reduced all-cause mortality among patients with a history of ischemic stroke, this finding should be interpreted cautiously. Given the observational nature of the study and the potential for residual confounding, we do not infer a direct protective effect of Oseltamivir on cardiovascular or cerebrovascular outcomes. Rather, it is more plausible that the observed association reflects the benefit of treating influenza itself, thereby reducing the risk of influenza-related complications and subsequent mortality. This interpretation aligns with the clinical understanding that influenza infection can exacerbate underlying conditions, particularly in patients with prior vascular events. Mouse models have demonstrated that influenza can exacerbate ischemic stroke outcomes via a cytokine cascade triggered by the virus [46]. Inhibiting cytokine release may mitigate ischemic brain damage and improve survival outcomes [46]. However, whether this mechanism fully explains the reduced mortality observed in our retrospective human study is uncertain. A study involving 1699 heart failure patients revealed significantly elevated plasma N-acetylneuraminic acid levels in 464 individuals who either died from cardiovascular-related causes or required heart transplantation, along with a marked increase in neuraminidase expression and desialylation in cardiac tissues [24]. Additionally, treatment with Oseltamivir in the heart failure mouse model notably reduced desialylation and improved cardiac dysfunction [24]. These findings suggest an alternative mechanism by which Oseltamivir might reduce mortality risk by mitigating cytokine-driven inflammation and addressing neuraminidase-mediated effects.

Despite statistical adjustment for age, sex, and comorbidities, several limitations warrant consideration. First, specific data on concomitant medications were not included in the analysis and could not be directly adjusted for. To partially account for this, we included major comorbidities in the multivariable models, which implies whether the patients had received certain treatments accordingly. Second, the database lacks information on socioeconomic status, ethnicity, and other demographic variables, which may lead to residual confounding. Third, Oseltamivir use is inherently tied to influenza diagnosis and clinical presentation, which may introduce treatment selection bias. Patients receiving Oseltamivir may differ from non-users in unmeasured ways, such as symptom severity, healthcare-seeking behavior, or physician prescribing patterns, which are not captured in claims data.

To further understand such an association, a more rigorous design, such as propensity score matching or restriction to a more epidemiologically homogeneous population without major comorbidities, would improve the validity of causal inference. Moreover, prospective cohort studies with detailed clinical and socioeconomic data or ideally randomized controlled trials are preferred to more definitively assess the impact of Oseltamivir on long-term outcomes of stroke incidence and mortality.

## 4. Materials and Methods

### 4.1. Data Source

The current investigation utilized a retrospective cohort design based on observational data, drawing data from the National Health Insurance Research Database (NHIRD), Ministry of Health and Welfare (grant no H110290). Established in 1995 with Taiwan’s NHI program, the NHIRD encompasses the entire population, covering outpatient and inpatient care, dental treatments, medications, and surgeries. As a result, the NHIRD stands among the world’s largest administrative health databases, being widely utilized for epidemiological and comparative effectiveness research published in major journals. To protect privacy, all data were anonymized; thus, informed consent was not required. The Chung Shan Medical University Hospital Institutional Review Board approved the study protocol (CS2-21180). Data access and all analyses were carried out at the Health and Welfare Data Science Center in Taiwan.

### 4.2. Patients with Oseltamivir Use

Oseltamivir use was defined based on prescription records following a confirmed diagnosis of influenza. According to national treatment guidelines, Oseltamivir is usually recommended in outpatient or emergency settings for patients with influenza that is clinically suspected or laboratory-confirmed in Taiwan. Therefore, the decision to prescribe Oseltamivir is largely driven by the presence of influenza symptoms and clinician judgment.

Patients were eligible for inclusion in the Oseltamivir group if they had a documented prescription for Oseltamivir between 1 January 2009 and 31 December 2018, were aged 20 years or older at the index date (defined as the first Oseltamivir prescription), and had no prior diagnosis of stroke (ICD-9-CM codes 430–438) before the index date.

To ensure data accuracy, several exclusion criteria were applied: individuals who had passed away before the index date, patients with duplicate entries, those under 20 years of age, individuals with unspecified gender, and any patients diagnosed with stroke (ICD9-CM codes 430–438) before the index date. Subjects under 20 years were excluded because pediatric patients require different Oseltamivir dosing and have substantially lower stroke risk. Hence, removing this population reduces potential bias due to heterogeneity. Moreover, age was adjusted for in the Cox model, so this criterion does not bias the results. Unspecified gender cases were excluded, since sex is a known stroke risk factor and is essential for covariate adjustment in the Cox regression model. Duplicate entries were removed to maintain data integrity and ensure accurate estimation of incidence and outcomes. Prior stroke history was excluded to focus on first-ever stroke events and avoid confounding from pre-existing cerebrovascular risk, as prior stroke strongly predicts recurrence. Death before the index date was excluded to prevent immortal time bias and ensure temporal accuracy in exposure–outcome relationships.

### 4.3. Control Group

Individuals who did not receive a prescription for Oseltamivir between 2009 and 2018 were selected for the control group. The index date was randomly assigned within the same study period (2009–2018) to approximate the temporal distribution of Oseltamivir prescriptions, ensuring comparable follow-up durations. The exclusion criteria for the control group were the same as those applied to the Oseltamivir user group.

### 4.4. Outcome Ascertainment

The study used the International Classification of Diseases, Ninth Revision, Clinical Modification (ICD-9-CM) codes to identify outcomes, specifically focusing on the relationship between Oseltamivir use and stroke (430–438) in Taiwan. To enhance the precision of the analysis, relevant comorbidities were also accounted for, including diabetes mellitus (250), hypertension (401–405), chronic renal insufficiency (585), chronic obstructive pulmonary disease (495–496), coronary artery disease (410–414), atrial fibrillation (427.31), hyperlipidemia (272.0–272.4), and peripheral artery disease (443).

### 4.5. Follow-Up

Participants were tracked from the date of index until the first stroke incident, death, or the end of the study period (31 December 2018), whichever occurred first. Those who died before receiving a stroke diagnosis were considered censored cases in the analysis.

### 4.6. Mortality Analysis

In addition, we aimed to examine how Oseltamivir exposure affects survival among patients with existing cerebrovascular disease. A separate cohort was constructed comprising individuals with a prior diagnosis of ischemic stroke (ICD-9-CM codes 433, 434, and 436) before the index date. This baseline stroke cohort was followed prospectively to assess differences in post-stroke mortality between Oseltamivir users and non-users. This design allows for the evaluation of survival outcomes among patients with established stroke history. Individuals who had passed away before the index date, patients with duplicate entries, those under 20 years of age, and individuals with unspecified gender were excluded.

### 4.7. Statistical Analysis

Continuous variables were reported as means and standard deviations (SDs), whereas categorical variables were presented as counts and percentages. Pearson chi-squared tests or independent sample *t*-tests were used to compare demographic differences between patients who took Oseltamivir and those who did not. The Kaplan–Meier method was utilized to estimate the cumulative incidence rate for Oseltamivir users and non-users. Cox regression analysis was conducted to calculate the hazard ratio (HR) for stroke development. Age, gender, and comorbidities were adjusted for. A two-tailed *p*-value < 0.05 was considered statistically significant. All data processing and statistical analyses were conducted using Stata 15 software (StataCorp, College Station, TX, USA).

## 5. Conclusions

In this study, we concluded that prescribing Oseltamivir in patients diagnosed with influenza is not associated with the incidence of stroke compared to those without Oseltamivir treatment. However, the use of Oseltamivir for influenza infection is highly recommended in patients treated either with a history of ischemic stroke or with potential dangers of stroke development due to its significant survival benefit.

## Figures and Tables

**Table 1:** Demographic information of patients.

	Total		Oseltamivir-Use Group		Non-Oseltamivir-Use Group		*p*-Value
Variable	N	%	N	%	N	%	
Total	13,676,072	100%	281,420	100%	13,394,652	100%	
Gender							<0.001
Male	6,323,529	46.2%	126,042	44.8%	6,197,487	46.3%	
Female	7,352,543	53.8%	155,378	55.2%	7,197,165	53.7%	
Age, years			40.1 ± 15.3		46.2 ± 16.0		<0.001
Comorbidities							
DM	1,157,457		15,729	5.59	1,141,728	8.52	<0.001
HTN	2,312,201		30,291	10.7	2,281,910	17.1	<0.001
CRI	188,386		3414	1.21	184,972	1.38	<0.001
COPD	349,389		9426	3.35	339,963	2.54	<0.001
CAD	504,781		7206	2.56	497,575	3.71	<0.001
AF	39,707		632	0.22	39,075	0.29	<0.001
Hyperlipidemia	1,344,577		19,047	6.77	1,325,530	9.9	<0.001
PAD	67,905		979	0.35	66,926	0.5	<0.001

AF, atrial fibrillation; CAD, coronary artery disease; COPD, chronic obstructive pulmonary disease; CRI, chronic renal insufficiency; DM, diabetes mellitus; HTN, hypertension; PAD, peripheral arterial disease.

**Table 2:** Comparison of ischemic stroke incidence and Cox proportional hazards analysis between Oseltamivir-use and non-Oseltamivir-use groups. HR, hazard ratio.

	Oseltamivir-Use Group	Non-Oseltamivir-Use Group	*p*-Value
Patients (n)	281,420	13,394,652	
Ischemic stroke incidence, n (%)	1314 (0.47)	93,707 (0.7)	
Cox analysis			
HR (crude)	0.62 (0.59–0.65)	1	<0.001
HR (adjusted for: sex + age)	1.13 (1.07–1.20)	1	<0.001
HR (adjusted for: sex + age + comorbidity)	1.02 (0.96–1.08)	1	0.511

**Table 3:** Comparison of mortality rates and Cox proportional hazards analysis between Oseltamivir users and non-users among patients with a history of ischemic stroke. HR, hazard ratio.

	Oseltamivir Use Group	Non-Oseltamivir Use Group	*p*-Value
Patients (n)	2502	221,499	
Mortality, n (%)	102 (4.08)	14,290 (6.45)	
Survival Cox analysis			
HR (crude)	0.72 (0.59–0.87)	1	0.001
HR (adjusted for: sex + age)	0.75 (0.62–0.92)	1	0.005
HR (adjusted for: sex + age + comorbidity)	0.74 (0.61–0.89)	1	0.002

## Data Availability

The data presented in this study are available on request from the corresponding author. The data are not publicly available due to privacy and ethical restrictions.
